# Insights into the adverse effects of prepubertal chronic ethanol exposure on adult female reproduction

**DOI:** 10.18632/aging.204851

**Published:** 2023-07-05

**Authors:** Qian-Nan Li, Guan-Mei Hou, Si-Min Sun, Wen-Bo Liu, Tie-Gang Meng, Yi Hou, Heide Schatten, Qing-Yuan Sun, Xiang-Hong Ou

**Affiliations:** 1 Fertility Preservation Lab, Reproductive Medicine Center, Guangdong Second Provincial General Hospital, Guangzhou 510317, China; 2 State Key Laboratory of Stem Cell and Reproductive Biology, Institute of Zoology, Chinese Academy of Sciences, Beijing 100101, China; 3 Department of Veterinary Pathobiology, University of Missouri, Columbia, MO 65211, USA

**Keywords:** alcohol, female, mouse, oocyte, prepuberty

## Abstract

Heavy drinking in women is known to adversely affect pregnancy and fertility. However, pregnancy is a complex process, and the adverse effects of ethanol on pregnancy does not mean that ethanol will have adverse effects on all stages from gamete to fetal formation. Similarly, the adverse effects of ethanol before and after adolescence cannot be generalized. To focus on the effects of prepubertal ethanol on female reproductive ability, we established a mouse model of prepubertal ethanol exposure by changing drinking water to 20% v/v ethanol. Some routine detections were performed on the model mice, and details such as mating, fertility, reproductive organ and fetal weights were recorded day by day after discontinuation of ethanol exposure. Prepubertal ethanol exposure resulted in decreased ovarian weight and significantly reduced oocyte maturation and ovulation after sexual maturation, however, normal morphology oocytes with discharged polar body showed normal chromosomes and spindle morphology. Strikingly, oocytes with normal morphology from ethanol exposed mice showed reduced fertilization rate, but once fertilized they had the ability to develop to blastocysts. RNA-seq analysis showed that the gene expression of the ethanol exposed oocytes with normal morphology had been altered. These results show the adverse effects of prepubertal alcohol exposure on adult female reproductive health.

## INTRODUCTION

Research on alcoholism is of wide concern because of its negative impact on many facets of life and physical health [1–5], including increased risk of infertility, seizures and other adverse reactions, and females are often shown to be more sensitive than males [6–8]. Previous studies have shown that female rats and mice were prone to have abnormal estrus cycles [9, 10], oocyte maturation ratio and ovulation number [11], after chronic ethanol intake. However, chronic ethanol exposure is known to result in abnormal development of some oocytes, but not all. There are still some oocytes that can discharge the polar body with normal morphology, and the status of these normal-shaped oocytes is uncertain. In addition, prenatal ethanol exposure can easily cause spontaneous abortion, premature birth, intrauterine growth retardation, and damage to the reproductive function and brain development of offspring [12–14]. The adverse effects of pre-pregnancy ethanol exposure on female fertility are widely known, and recognized as temporary, not permanent, however, no direct studies have been able to provide theoretical support.

In prepubertal female ovaries, primordial follicles initiate growth to primary follicles and grow gradually, but they go to atresia before the antral follicle stage due to the lack of gonadotropins. After estrus, some follicles gradually mature and excrete MII-stage oocytes, following the change of gonadotropin secretion in waves. The developmental potential of oocytes before puberty is lower than that of oocytes from sexually mature individuals [15, 16]. To further understand the effects of prepubertal ethanol exposure on reproduction of sexually mature females, we established a mouse model of chronic prepubertal ethanol exposure by changing drinking water to 20% v/v ethanol for three weeks, and examined oocyte quality, reproductive capacity, and offspring status. These experiments were expected to further define the dangers of alcohol consumption during adolescence.

## MATERIALS AND METHODS

### Prepubertal ethanol exposure in a female mouse model

Three-week-old C57BL/6J female mice were purchased from SPF (Beijing) Biotechnology Co., Ltd. The mice were raised in cages of five and divided into two groups. One was the control group (CtrL), the other was the 3-week ethanol exposure group (EtOH) by replacing drinking water with 20% v/v ethanol.

### Glucose tolerance test and insulin resistance test

The blood glucose of all mice was measured, and the mean-values of blood glucose of CtrL- and EtOH- groups were calculated, respectively. The mice with blood glucose levels near mean-value were selected for testing. For glucose tolerance test (GTT) and insulin resistance test (ITT), mice were fasted for 16 h and 6h, respectively, then 2 g/kg glucose or 1 IU/kg insulin was injected in the abdominal hypodermis. Tail blood was collected for blood glucose detection and blood glucose was measured 0 min, 15 min, 30 min, 45 min, 60 min, 90 min and 120 min after injection by a glucometer (Roche).

### Estrous cycle detection

After a 3-week treatment, mice were evaluated for estrous cycles daily for 18 days at the same time by vaginal lavage. The first 6 days of vaginal lavage were the time of acclimation for the mice, the other 12 days of vaginal lavage were used for smears. The smears were observed under a microscope, and classified as proestrus (P), estrus (E), metestrus (M) and diestrus (D) through the proportion of nucleated epithelial cells, cornified squamous epithelial cells and leukocytes [17].

### Oocyte maturation, fertilization and embryonic development

The processes followed published protocols [18]. The ovaries were removed with tweezers and weighed, then chopped up in M2 medium (Sigma, USA). The GV-stage oocytes were selected microscopically and matured *in vitro* in M2 medium. The number of germinal vesicle breakdown (GVBD) oocytes was recorded after 2-4 hours and the number of oocytes that extruded polar bodies (PBE) was recorded after 14 hours. Forty-eight hours after i.p. injection with pregnant mare serum gonadotrophin (Ningbo, China), hCG (Ningbo, China) was injected. Fourteen hours later, cumulus-oocyte complexes (COCs) were collected from the Fallopian tubes. For IVF, spermatozoa were obtained from healthy adult male mice and kept in HTF (Merck, Germany) for 40 min. Sperm and COCs were incubated in HTF. After 10-20 minutes of incubation, MII-stage oocytes with normal morphology were selected microscopically and then co-incubated with spermatozoa for 4-6h in HTF. After sperm-oocyte co-incubation, embryos were cultured in KSOM (Millipore, Germany) until development to blastocysts *in vitro*. The fertilization rates were determined by counting the number of pronuclei observed 8-9 hours post-fertilization. The number of blastocysts was confirmed at 4.5dpc and 5.5dpc respectively. In addition, female mice with vaginal plug after mating with male were used to observe blastocysts *in vivo* at 3.5 dpc by uterus flushing.

### Fertility determination

After three weeks of treatment, the female mice were discontinued ethanol exposure, and mated with healthy adult males for 5 days. The dates of females secreting vaginal plugs were recorded. The pregnancy in the mice with vaginal plugs was recorded to calculate the fertility rate, and fetuses and placentas were collected intact using scissors and forceps for weighing at 19.0 dpc.

### Chromosome spreading

The processes were performed as described previously [19]. MII-stage oocytes matured *in vivo* were collected 14 hours later from the Fallopian tubes, and digested with 1 mg/ml hyaluronidase (Sigma, USA). The zona pellucida of MII-stage oocytes matured *in vivo* were removed with acid Tyrode’s solution (Sigma, USA). The oocytes without zona pellucida were fixed in 1% paraformaldehyde with 0.15% Triton X-100 and 3 mM dithiothreitol on glass slides. The slides were dried at room temperature and blocked with 1% BSA for 1 h, then incubated in anticentromere antibodies, (ACA; 1:50) at 4° C overnight. After washing, the slides were incubated with secondary antibodies Cy5 (1:200) for 1 h at room temperature. The nuclei were stained with DAPI at room temperature for 15 min. DABICO was added and coverslips were used for lightly covering the samples.

### Immunofluorescence

MII-stage oocytes were collected and fixed in 4% paraformaldehyde for 20 min. After incubation in 0.1% Triton X-100 for 20 min, the oocytes were transferred to 1 mg/ml BSA for 1 h. The processes above were performed at room temperature. Then the oocytes were incubated with α-tubulin antibody at 4° C overnight, and nuclei were stained with DAPI. Finally, samples were placed on the slides and DABICO was applied. All the specimens were observed under a Zeiss LSM 780 confocal laser-scanning microscope.

### RNA-seq

RNA-seq was performed by Annoroad Gene Tech. (Beijing) Co., Ltd. The MII-stage oocytes were collected by superovulation from 3-week-old mice, 6-week-old control mice and ethanol-exposed mice in tubes with lysing reagent (30 oocytes per tube). All the collected oocytes had obviously extruded polar bodies. Ethanol-treatment did not stop during superovulation in the ethanol-exposed group. Amplification was conducted by the Smart-Seq2 method. After library preparation, PerkinElmer LabChip GX Touch and Step OnePlus Real-Time PCR System were introduced for library quality inspection, and PE150 sequencing was performed using the Illumina HiSeq platform. The experiments of each group were repeated three times. The RNA-seq data were available under NCBI SRA accession number PRJNA719111.

### Statistical analysis

Data from repeated experiments were analyzed by independent sample T test by SPSS software. P<0.05 was considered a significant difference. The area under the curve (AUC) of GTT and ITT were calculated with GraphPad Prism version 6.01 for windows, GraphPad Software, La Jolla California USA. Correlation analysis was performed on the gene expression levels of each group by SPSS.

## RESULTS

### Establishment of prepubertal chronic ethanol exposure mouse model

**Figure 1:**
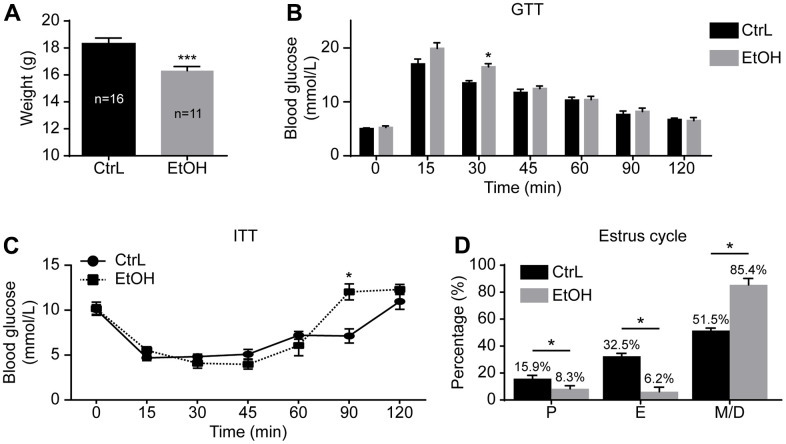
**Establishment of prepubertal chronic ethanol-exposed mouse model.** (**A**) Body weight. (**B**) Blood glucose levels in glucose tolerance test (GTT). (**C**) Blood glucose levels in insulin resistance test (ITT). (**D**) Estrous cycle. P, proestrus, E, estrus; M/D, metestrus and diestrus; *, p<0.05. ***, p<0.001.

To understand the effect of prepubertal chronic ethanol exposure on adult reproduction, we established a prepubertal ethanol exposure female mouse model by changing the 3-week-old mice’s drinking water to 20% v/v ethanol for 3 weeks. After a 3-week ethanol treatment, mice went through prepuberty, and the weight of ethanol-treated mice (n=11) was significantly lower than that of the control mice (n=16) (Figure 1A).

The GTT and ITT were also performed to make comparison between the control and ethanol-exposed groups. GTT showed that the glucose level at 30-min post-injection was significantly higher in ethanol-treated mice (16.13 ± 0.6438 mmol/L vs 13.50 ± 0.4359 mmol/L, p<0.05; n=6 vs 6) (Figure 1B), and ITT showed that the glucose level at 90-min post-injection of insulin was also significantly higher in ethanol-treated mice (12.03 ± 0.8950 mmol/L vs 7.133 ± 0.7881 mmol/L, p<0.05; n=6 vs 6) (Figure 1C). Although blood glucose of GTT and ITT changed at individual time points, the trend of change was not significant. The area under the curve (AUC) of GTT (1235 ± 52.12 mmol/L/min vs 1299 ± 22.26 mmol/L/min, p>0.05) and ITT (835.5 ± 42.11 mmol/L/min vs 962.3 ± 58.14 mmol/L/min, p>0.05) of both groups were almost the same. The peak value and the corresponding time point of both groups showed no significant difference on GTT and ITT. The risks of prepubertal ethanol exposure cannot be measured by blood sugar. In other words, the absence of abnormal blood sugar levels does not mean that alcohol is not harmful to health.

To further understand the state of prepubertal ethanol exposed mice, the estrus cycle detection was carried out after 3-week of ethanol exposure by vaginal smears. Vaginal smears were examined for 18 consecutive days. The first 6 days of vaginal lavage were the time of acclimation for the mice, the other 12 days of smears were used to record the estrus cycle of model mice (n=17) and control mice (n=16). The results showed that the prepubertal ethanol-exposed female mice had a disordered estrus cycle, and most of the time the ethanol-exposed mice were in metestrus and diestrus (Figure 1D). This suggested that prepubertal chronic ethanol exposure might have adverse reproductive effects in adults.

### Effects of prepubertal chronic ethanol exposure on adult female fertility

**Figure 2:**
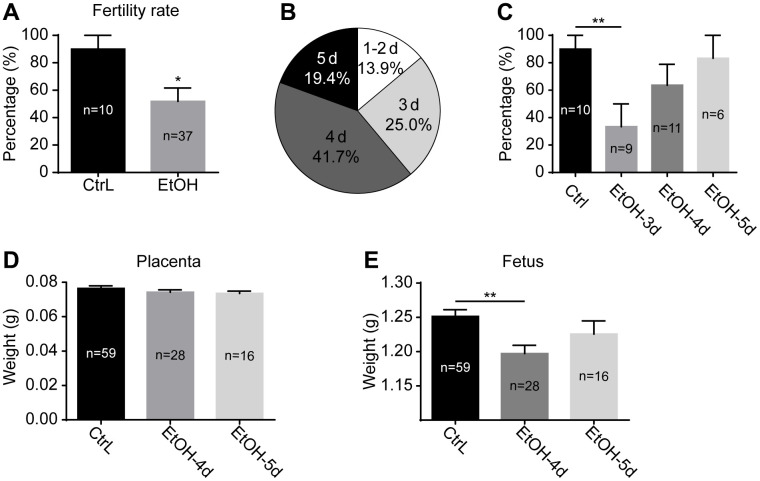
**Effects of prepubertal chronic ethanol exposure on fertility.** (**A**) the fertility rate of prepubertal ethanol-exposed female mice. (**B**) The distribution of successful mating dates after discontinuation of ethanol exposure. (**C**) The fertility rate of prepubertal ethanol-exposed mice from different successful mating dates. (**D**) Placenta weight of prepubertal ethanol-exposed mice from different successful mating dates. (**E**) Fetus weight of prepubertal ethanol-exposed mice from different successful mating dates. *, p<0.05; **, p<0.01.

After a 3-week ethanol exposure, the mice reached puberty. The ethanol exposure was stopped immediately, and the mice were mated with normal adult males. Females were observed daily for the presence of vaginal plugs, and mice with vaginal plugs were recorded as successful mating. The production of offspring was recorded as being fertile. The results showed that the fertility rate of prepubertal ethanol-exposed female mice decreased significantly compared with control mice (Figure 2A). After discontinuation of ethanol exposure, the distribution of successful mating dates was shown in Figure 2B. A day or two after abstaining from ethanol, fewer mice mated successfully and produced few offspring. The fertility rate of the successfully mating mice on day 3 after abstaining from ethanol was significantly lower than that of the control mice, and that of day 3 and 4 showed no significant difference between the two groups (Figure 2C). Weight of placentas collected at 19.0 dpc showed little difference in prepubertal ethanol-exposed mice that mated successfully on day 4 and 5 compared to the control mice (Figure 2D). Fetal weight of prepubertal ethanol-exposed mice mating successfully on day 4 was significantly lower than that of the control mice, and the difference was eliminated at day 5 (Figure 2E). From this, we inferred that ethanol has an adverse effect on mouse reproduction, and timely withdrawal of ethanol exposure restore fertility to some extent. It was unknown whether the adverse effect on mouse reproduction occurred only during fertilization and embryonic development or began at the oocyte stage.

### Prepubertal chronic ethanol exposure leads to a decrease in oocytes of adult mice

**Figure 3:**
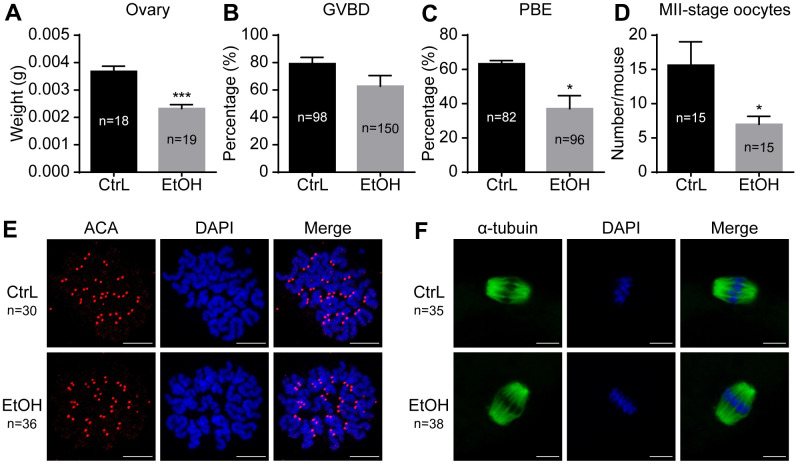
**Effects of prepubertal chronic ethanol exposure on ovary and oocytes.** (**A**) Ovary weight. (**B**) Germinal vesicle breakdown (GVBD) rate. (**C**) Polar body extrusion (PBE) rate. (**D**) Number of MII-stage oocytes *in vivo*. (**E**) Chromosome spreading in MII-stage oocytes matured *in vivo*. (**F**) Spindle stained in MII-stage oocytes matured *in vivo*.

To understand the effects of prepubertal chronic ethanol exposure on female reproduction, the examination of oocyte number and quality of prepubertal ethanol-exposed mice is necessary. The results showed that the ovarian weight of prepubertal ethanol-exposed mice was significantly reduced (Figure 3A). *In vitro* culture of GV-stage oocytes taken from ovaries showed no significant difference in GVBD rates, but the PBE rate was significantly reduced (Figure 3B, 3C). The number of normal MII-stage oocytes collected by superovulation from each prepubertal ethanol-exposed mice (n=15) was significantly lower than that of the control group (n=10) (Figure 3D). Therefore, we inferred that ethanol feeding leads to abnormal growth and development of ovary and reduces the number of oocytes.

### Prepubertal ethanol exposure causes reduced fertilization ability of morphologically normal adult oocytes, but once oocytes fertilized have the ability to develop into blastocysts

**Figure 4:**
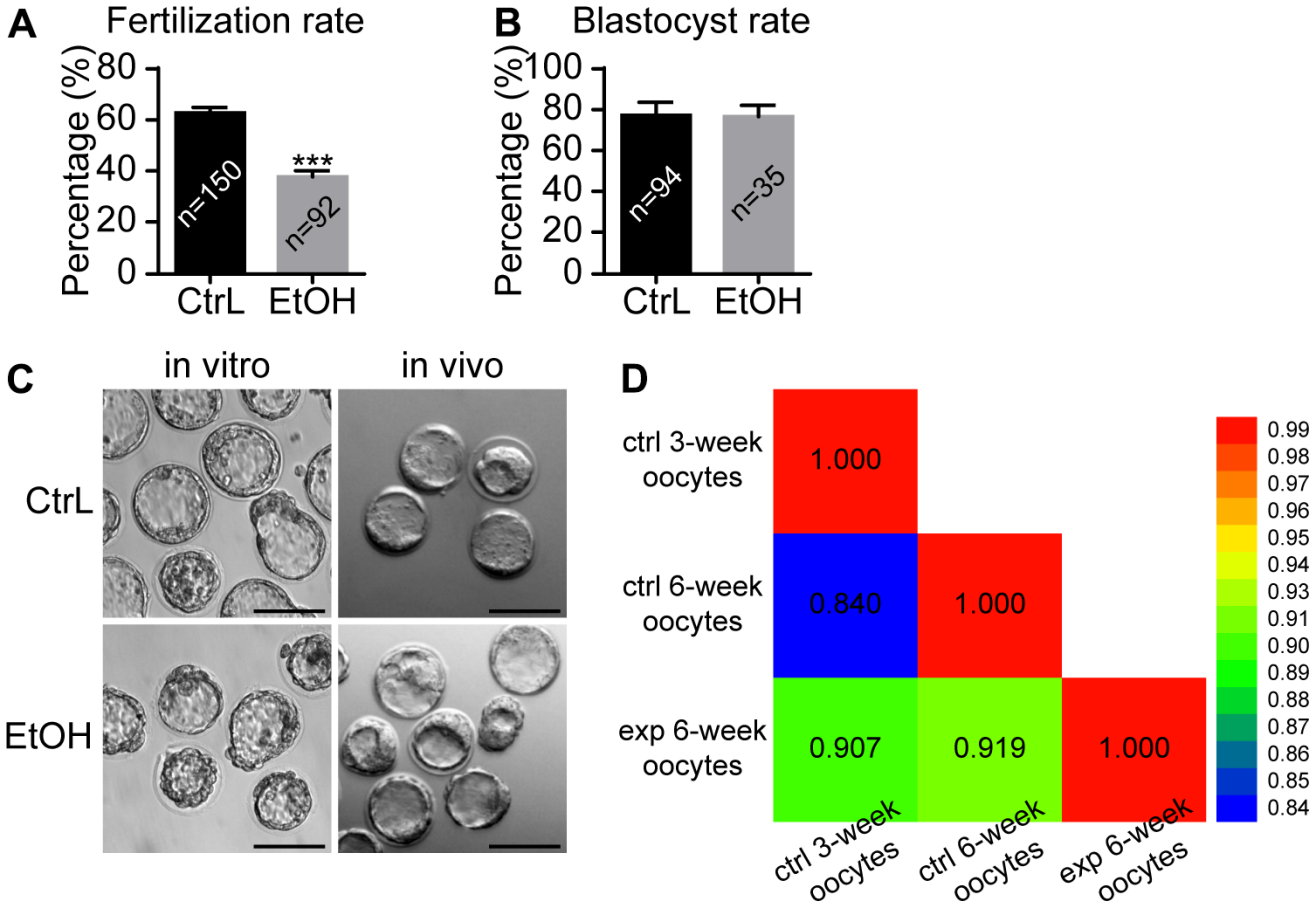
**Effects of prepubertal chronic ethanol exposure on fertilization ability.** (**A**) Fertilization rate after *in vitro* fertilization. (**B**) Blastocyst rate from zygotes. (**C**) The morphology of blastocysts produced *in vitro* and *in vivo*. ACA, anticentromere antibodies; *, p<0.05; ***, p<0.001. (**D**) Bivariate analysis between any two of CtrL-3-weeks, CtrL-6-weeks, and EtOH-6-weeks.

It was reported that oocytes exposed to ethanol solution might show chromosome separation abnormalities by affecting the spindle [20, 21]. In our study, ethanol exposure caused abnormal morphology in some oocytes, while the other oocytes still showed normal morphology whose status remained uncertain. Immunostaining showed that spindles were basically normal and the separation of chromosomes was normal in morphologically normal oocytes with extruded first polar body (Figure 3E, 3F). We next clarified whether ovulated oocytes with normal morphology from ethanol exposed mice have normal developmental potential. IVF was performed for MII-stage oocytes with normal morphology obtained by superovulation. Prepubertal ethanol-exposed groups showed a significant decrease in the fertilization rate (Figure 4A), but no significant difference in blastocyst rate was observed when counting from zygotes to blastocysts (Figure 4B). After superovulation, both *in vitro* and *in vivo* matured oocytes had the ability to produce normal blastocysts (Figure 4C). This suggested that oocytes with normal morphology from ethanol exposed mice, once fertilized, could develop into blastocysts, although prepubertal ethanol exposure had a negative effect on the maturation and fertilization of oocytes.

### RNA-seq for ethanol exposed oocytes with normal morphology

**Figure 5:**
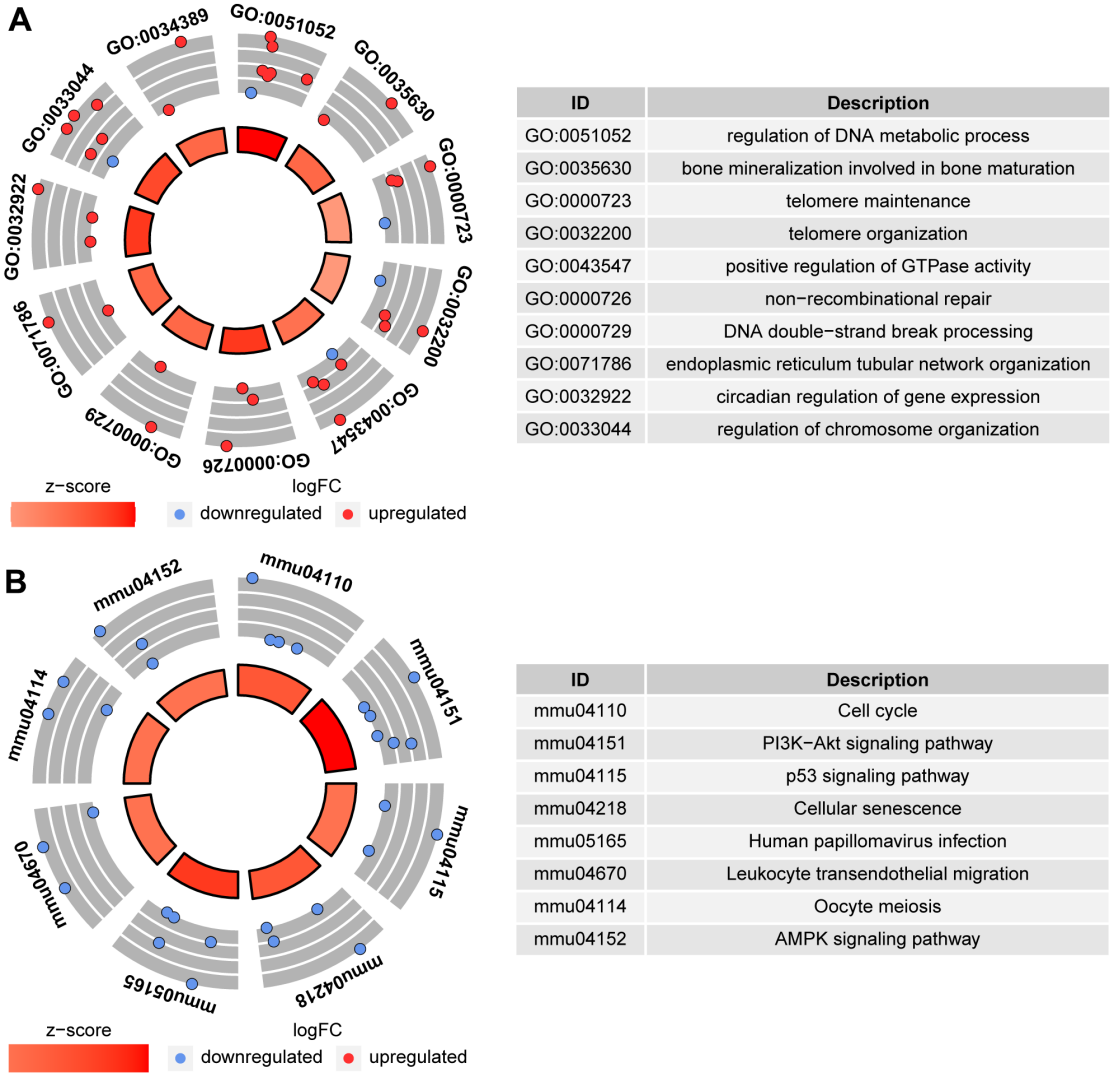
**GO analysis and KEGG analysis between CtrL-6-week-group and EtOH-6-week-group.** (**A**) GO analysis. (**B**) KEGG analysis.

To determine the quality of the oocytes with normal morphology after ethanol exposure during the period from 3 to 6 weeks after birth, RNA-seq was performed on MII-stage oocytes from control mice (CtrL-6-week) and the oocytes with normal MII-stage morphology from ethanol exposed mice (EtOH-6-week). Comparing CtrL-6-week-group and EtOH-6-week-group, GO analysis showed that there were 234 GO terms which were significantly enriched (P<0.05, Figure 5A). KEGG analysis showed that there were 8 KEGG pathways which were significantly enriched (P<0.05, Figure 5B). Among them, topping the list of KEGG pathways is “Cell cycle” pathway. The results showed that alcohol had an adverse effect on oocytes related to cell cycle, meiosis and cell senescence and other factors.

The GO and KEGG analysis showed that the state of oocytes was changed by ethanol treatment, but the intensity of the change was difficult to predict. To understand the damage caused by 3 weeks of ethanol treatment, RNA-seq was performed on oocytes from normal female mice (CtrL-3-week) at 3 weeks of age without ethanol treatment and sexual maturation. Bivariate analysis was used to analyze the correlation between any two of CtrL-3-week, CtrL-6-week, and EtOH-6-week (Figure 4D). Some studies have shown that the developmental potential of oocytes before puberty was lower than that after sexual maturation [15, 16], this explained the relatively low correlation coefficient between CtrL-3-weeks and CtrL-6-weekss (Pearson coefficient = 0.840). The correlation coefficient between CtrL-3-weeks and CtrL-6-weeks was lower than that between EtOH-6-weeks and CtrL-6-weeks (Pearson coefficient = 0.919), suggesting that the damage to morphologically normal oocytes after ethanol treatment was weaker than the lack of developmental potential caused by insufficient sexual maturation. In addition, The correlation coefficient between CtrL-3-weeks and CtrL-6-weeks was lower than that between EtOH-6-weeks and CtrL-3-weeks (Pearson coefficient = 0.907). It could be speculated that there might be some correlation between ethanol injury and the loss of developmental potential without experiencing sexual maturation. These results might help us to understand the extent of oocyte damage caused by ethanol.

## DISCUSSION

Alcohol exposure has adverse effects on the reproductive system of females and males [22, 23], and can also cause abnormal preimplantation embryo development. In our current society, more and more preadolescent women are inevitably exposed to alcohol [24]. Therefore, it is necessary to explore the reproductive effects of preadolescent alcohol exposure. We first constructed a mouse model of prepubertal chronic ethanol exposure and found that after 3 weeks of prepubertal ethanol exposure, mice exhibited significantly reduced body weight gain and disturbed estrus cycles. Studies have shown that the content of estrogen in the blood was different in different estrus phases, and alcohol had different effects on different estrus phases of female mice [9]. Estrogen was involved in the process of bioenergy imbalance caused by ethanol [25, 26]. Prenatal ethanol exposure could lead to menstrual disorders [12]. Our mouse model further proved that prepubertal ethanol exposure can lead to a disordered estrus cycle of adult mice. The fertility rate of mice that mated successfully on day 1 to 3 after discontinuation of ethanol decreased significantly, but gradually recovered after day 4. Fetal weight at 19.0 dpc was significantly lower in mice successfully mated 4 days after ethanol withdrawal but recovered in mice mated 5 days after ethanol cessation. These results showed that the adverse effect of ethanol on fertility can be relieved with the cessation of ethanol.

In addition, prepubertal ethanol exposure affects the growth and development of ovaries and maturation of oocytes. In spite of this, some oocytes with normal morphology could be obtained from ethanol exposed mice. These oocytes had normal chromosomes and spindles. *In vitro* fertilization of MII-stage oocytes of prepubertal ethanol exposed mice showed that the fertilization rate decreased significantly but once fertilized, the zygotes developed normally into the blastocysts *in vitro*. In addition, we were able to obtain normal blastocysts *in vivo* from prepubertal ethanol-exposed mice. Oocytes with normal morphology from ethanol exposure mice were affected in terms of binding with sperm, while fertilized eggs could develop into blastocysts. In addition, our RNA-seq analysis showed that the status of the oocytes with normal morphology after ethanol exposure had been altered, although not dramatically. If ethanol had a serious impact on oocytes, it would not be possible to restore fertility in a short period of time, since the oocyte growth takes a relatively long time. Lack of dramatical damage provided a prerequisite for the gradual fertility restore to normal in 4 days from discontinuation of ethanol exposure. There were some differences with previous results. Previous studies had shown that ethanol exposure *in vivo* lead to very poor oocyte quality, because these studies did not distinguish between morphologically normal and abnormal oocytes. This study focused on oocytes with normal morphology after ethanol treatment, which led to a difference in the results.

Follicles undergo initial recruitment and cyclic recruitment during their growth. The initial recruitment begins after the mouse is born, and the cyclic recruitment refers to the process by which follicles respond to changes in gonadotropins during the estrus cycle after puberty. The developmental potential of oocytes before puberty was lower than that after sexual maturation. The estrus cycle of prepubertal ethanol exposure mice was disordered, and the disorder mainly appeared as stagnation in metestrus and diestrus period and was basically not in estrus period. This state of estrus disorder indicated that female mice did not have the ability to initiate cyclic recruitment. We suspected that ethanol exposure caused the retardation of reproductive development of prepubertal female mice. That was, after 3 weeks of ethanol-treatment, even if the mice have reached adult age, their physical condition was still not ready to initiate follicle cyclic recruitment. In other words, the state of oocytes from prepubertal ethanol-exposed mice should be between a poor state of prepuberty and a healthy state of adulthood.

In fact, this concept was difficult to verify. In addition, the effect of ethanol on prepubertal females was not only developmental delay, but there were still other damages as well. We aimed to using RNA-Seq and bivariate analysis to determine the correlation between oocytes from prepubertal, adult, and ethanol-exposed mice. Notably, we found that the correlation coefficient between CtrL-3-week and CtrL-6-week was lower than that of the two other combinations. That was to say that there might be some correlation between ethanol injury and the loss of developmental potential without experiencing sexual maturation. So, it was concluded that prepubertal chronic ethanol exposure resulted in delayed oocyte development.

Our ethanol exposure protocol was to replace the daily drinking water of mice with 20% ethanol. This method involved less human intervention in mice compared with other operational schemes such as injection and gavage which causes emotional stress including fear and resistance. Therefore, the obtained reproductive results in the present study could be more reliable. Cebral et al. defined 5% as low chronic ethanol consumption and 10% as moderate doses of ethanol, while suggesting that 10% could result in many poor eggs [22, 27]. In order to highlight the phenotype enough, we increased the concentration of ethanol in the hope that we could tell whether the oocyte with normal morphology was a healthy oocyte. Therefore, the final concentration of ethanol was set at 20%.

Our study showed that prepubertal chronic ethanol exposure affected female fertility, but it was possible to select good quality MII-stage oocytes by morphology. Studies had shown that different genetic backgrounds produce different individual differences in alcohol consumption [28], so our conclusions might differ across species. In any case, our study did not recommend excessive drinking before puberty. Drinking often leads to the disorder of a number of physical indicators in the body, which would have an impact on reproduction. Our study provided evidence of adverse effects of prepubertal alcohol consumption on reproductive health of adults.
